# Brands and Inhibition: A Go/No-Go Task Reveals the Power of Brand Influence

**DOI:** 10.1371/journal.pone.0141787

**Published:** 2015-11-06

**Authors:** Nicholas Peatfield, Joanne Caulfield, John Parkinson, James Intriligator

**Affiliations:** 1 School of Psychology, Bangor University, Bangor, Wales, LL57 2AS, United Kingdom; 2 Center for Mind/Brain Sciences, University of Trento, Rovereto (TN), Italy; University of Bologna, ITALY

## Abstract

Whether selecting a candy in a shop or picking a digital camera online, there are usually many options from which consumers may choose. With such abundance, consumers must use a variety of cognitive, emotional, and heuristic means to filter out and inhibit some of their responses. Here we use brand logos within a Go/No-Go task to probe inhibitory control during the presentation of familiar and unfamiliar logos. The results showed no differences in response times or in commission errors (CE) between familiar and unfamiliar logos. However, participants demonstrated a generally more cautious attitude of responding to the familiar brands: they were significantly slower and less accurate at responding to these brands in the Go trials. These findings suggest that inhibitory control can be exercised quite effectively for familiar brands, but that when such inhibition fails, the potent appetitive nature of brands is revealed.

## Introduction

Within the consumer environment there is an abundance of products and brands from which a consumer can choose. For example, when faced with the option of selecting a new television, does one choose plasma or LCD? Once the kind of television is chosen, will one buy a Sony, a Samsung, or any of the numerous other brands? During each stage of the consumer decision-making process there are many options from which to choose, and when choosing one option the others must be inhibited. Inhibition is thus an integral part of selection and decision-making. Within the consumer domain, the impact of selection and inhibition has been studied in relation to memory processes: for example, unchosen (and thus inhibited) brands are more difficult to recall [1–4] and memory for inhibited advertisements tends to be worse [5]. Unlike these previous studies that examined cognitive responses to inhibition, the current study is concerned with action-based inhibitory control. More specifically, we are interested in understanding whether brand logos evoke differential effects on inhibitory control as a function of brand familiarity and liking. We have chosen to examine these two factors (familiarity and liking) because they are two of the principle factors thought to contribute to the ‘power’ of brands [6].

### Inhibitory Control

In its most basic form, “inhibitory control” simply refers to the action of inhibiting a pre-potent desire or tendency to respond. One common way to study inhibitory control is by building up a habitual response pattern in participants and then intermittently requiring them to withhold this response on cue. This is the basic logic within the Go/No-Go (GNG) paradigm that we employ here [7]; see Think/No-Think for another example [8]. In the GNG paradigm, 90% of the trials require participants to respond (e.g. press the space bar). This creates a pre-potent tendency to respond. However, on the No-Go trials (10% of trials), a signal is given (e.g. the stimulus is repeated) and the participant must withhold responding. Withholding a response requires an individual to override the pre-potent response and hence performance on this task can provide a behavioral assay of inhibitory control.

Because performance on GNG depends both on the individual doing the responding and on the stimulus eliciting a response, the task can be used to assess both. Thus, not only can this task measure behavioural inhibition, but it can also be used to explore the interaction between affect and cognition. For example, by employing affective stimuli (or testing participant groups with characterized affective disorders) the task can assess whether particular stimulus properties can influence the self-control of individuals [9,10]. Following this logic, Murphy and colleagues [11] found that manic patients were faster to respond to happy stimuli and that depressed patients were quicker to respond to unhappy stimuli. Similar results have been reported with error data in depressed patients [12]. In addition, a GNG study using affective facial expressions found participants responded more slowly to frightened faces and found it difficult to inhibit responses to happy faces[13].

Another important concept is the idea of the two motivational systems of approach and avoid [14]. Chen and Bargh [15] applied this concept by using a experimental procedure that measured reaction times to pushing and pulling levers, participants were shown to push away negative valance stimuli quicker than they would pull, and the opposite for positive valance stimuli (see review, [16]). Within the context of inhibitory control the response towards stimuli can be seen to be driven by these underlying mechanisms, and that the most likely is an approach mechanism guiding commission errors especially driven by exercising self-control [17].

#### Liking and Familiarity

Within the marketing domain there is known to be a relationship between how familiar one is with a brand and how much the brand is liked [6]. Specifically, more familiar brands tend to be more liked. Rindfleisch & Inman [6] suggest that this relationship is driven by the social desirability of the item.

More recently, this relationship has been explained based on perceptual fluency. Specifically, the logic goes something like this: things we like tend to get processed more rapidly by our brain; as one becomes more and more familiar with a brand it becomes easier to “process” the brand (visuo-cognitively); consequently, this increased perceptual fluency gets mis-interpreted as indicating a greater degree of “liking” of the brand [18]. This “mere exposure” effect has been demonstrated within the context of advertising, and can occur without conscious recollection of the item [19]. Simultaneously, there is evidence that, in certain context, item familiarity can lead to a reduction of liking, for example stimuli that are irrelevant to the task demands and are actively inhibited develop what [20] coin “distractor devaluation”.

Whatever the cause of the familiarity-liking relationship, it is clear that these two concepts are deeply intertwined. And, that this is likely particularly true for brands, which will have widely varying levels of liking and familiarity. Thus, when trying to study the impact of brands on inhibitory control (or any cognitive, affective, or motor faculties), it is important to measure stimuli for both liking and familiarity.

#### Brands and Cognitive Measures

There is a growing body of literature that looks at the influence of brands on cognitive tasks, and indeed this influence has been used as a measure of consumer perception towards marketing [21]. One of the most commonly used tasks to measure the “unconscious” attitudes of consumers’ is the implicit association test (IAT; [22]) which measures the reaction times towards a stimulus, and the cost of switching categories. Using this task, and the somewhat related Go/No-go Association Task (GNAT; [23]), researchers have measured the divergence of explicit consumer preference and implicit consumer preference [24,25]. These studies, and others, highlight the ability for cognitive tasks, and in particular reaction time measures, to quantify the influence of brands and of consumer preference.

Indeed, it is this previous literature that demonstrated the impact of consumer preferences on cognitive task performance that allows us to make inferences about the outcome of the GNG related to brand stimuli. For example, a study Thomas, Hammer, Beibst, and Münte [26] investigated the response-times within a GNAT task for brand stimuli, and they demonstrated faster response for brand products compared to unbranded products, and the authors highlight how neurophysiological measures can also be employed to understand implicit attitudes. Although the GNAT and the GNG are closely related it must be noted that they differ in that the no-go in the GNAT is a category whereas the no-go in a GNG is a signal (e.g. a stimulus repetition).

#### Aims of present study

Previous research has used the GNG paradigm to understand aspects of basic behavioural control in normal and clinical populations. More recent studies have investigated the effects of affectively or motivationally relevant stimuli on participant performance (for example, [9,27]). The current study examines affective influences (liking and familiarity) on inhibitory control by employing brand stimuli (logos) which participants rate for both familiarity and liking. Due to individual differences in brand familiarity and liking, we expected to find a large range of liking and familiarity across the set of brands. We predicted that there would be an overall effect of familiarity, shown by a faster response to familiar brands than unfamiliar brands. Furthermore, we predicted that there would be an effect of subjective liking towards the brands evidenced by a response tendency (e.g. increased failures to inhibit responses) towards liked vs non-liked brand stimuli, as these brands will evoke approach errors of inhibition as seen before within the context of valance [13].

## Method

### Participants

Twenty-eight undergraduate psychology students (15 Female, mean age 20.1) from Bangor University volunteered to participate through an online experimentation booking system. The entire procedure took approximately 30 minutes and participants received course and printer credits for their participation.

### Ethics Statement

The research was approved by the School of Psychology, Bangor ethics committee, and was done in according to the principles expressed in the Declaration of Helsinki.

### Apparatus

E-Prime experimentation software (PST, 2002) was used to conduct both parts of the experiment. The software ran on Pentium 4 (3.06 GHz) computers running the Windows XP operating system. The experiment was displayed on 17” CRT monitors (800x600, 85Hz, 32bit). Participants sat in front of the monitor at an approximate distance of 55 cm.

### Stimuli

120 brand logos were used throughout the tasks. Logo selection was based on previous results obtained at the lab (dozens of rating studies have given us a database of familiar and liked brand logos) and also on our intuition as to which brands would be unfamiliar to UK students. Our *a priori* classification of brands as “familiar” or “novel” was subsequently confirmed by participant ratings (as discussed below). 60 of the brands were familiar and included a range of brand categories (e.g: Electronic Consumer Goods: Apple, Sony, Samsung; and Toiletries: Colgate, Crest; amongst others) and 60 of the brands were unfamiliar. The physical dimensions of the brand logo stimuli were 320 x 320 pixels.

### Measures

During the first (GNG) phase, participant performance was measured in terms of response times and accuracy. During the second (rating) phase the participants were asked two questions about the brand logos: “How familiar are you with this brand?” and, “How much do you like this brand?” They responded to these questions on a five-point Likert scale [28]. ranging from one (“negative”), through three (“neutral”), to five (“positive”).

### Procedure

**Fig 1:**
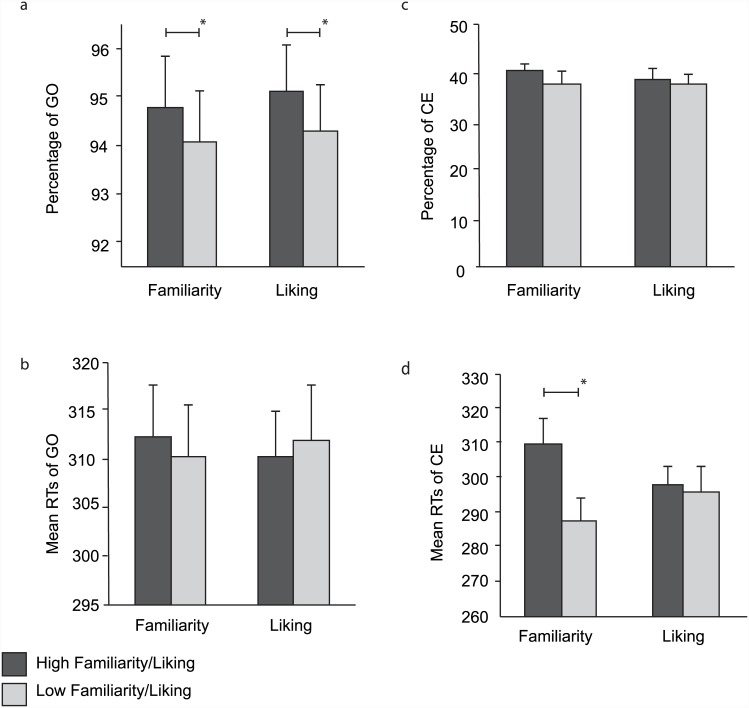
Performance of participants for the different measures, and the different subjective-ratings. (a) GO trial accuracy. I.e. pressing the space bar when required to do so. (b) Percentage of commission errors. I.e. pressing the space bar when it was not required to do so. (c) Mean RT for GO trials, (d) Mean RT for commission errors. Error bars indicate standard error. * Denotes significance.

After a brief presentation on the requirements during experimentation, the participants were given an information sheet and consent form, which they were asked to complete before continuing. Participants were then asked to complete the GNG task. They were informed that they needed to respond as quickly as possible when brand logos were presented on the screen (Go trials), and not to respond to a brand logo if it repeated itself (No-Go trials). The required response to the Go trials was the spacebar, and participants were asked to place both index fingers on it. The GNG trial frequency was 1 Hz (see Fig 1). Within the 1000 msec trial window the brand logo was presented for the first 600 msec, and the remaining 400 msec was a blank screen. During the ISI the screen was black. During the logo presentation period the logo took up the centre 320x320 pixels of the screen and the rest of the screen was the same black as in the ISI. The Go/No-Go experiment consisted of 1080 trials broken into four blocks of 270 trials, this lead to 8 presentations of each brand as a Go trial and a single presentation as a No-Go (repeat stimulus) trial. At the end of each block participants had a self-timed break (minimum of 30 sec).

After the GNG task participants started the rating task in which they were asked to rate all stimuli for familiarity and liking (in separate blocks). Participants were informed that their ratings should be based upon their knowledge/experience *before* the GNG task: that is, simply seeing a brand logo in the preceding GNG task should not be enough to make it count as being familiar. The close correlation between our *a priori* sub-set of unfamiliar brands and participant ratings suggest that this was indeed the case. A difference score was calculated and a Ttest against 0 was performed, p = 0.001, Unfamiliar: M = 1.7, SD = 0.5; Familiar M = 4.15 SD = 0.7. Participants always completed a block of familiarity ratings first followed by a block of liking ratings. Each trial rating presented the brand image in the center of the screen with a text prompt displayed at the bottom of the screen showing the scale (1–5), guide words (at 1, 3, and 5), and the optional key responses. Upon completion of the rating tasks (familiarity and liking) participants were thanked and then debriefed about the study. The Go/No-Go task lasted about 20 minutes and the rating task lasted about five minutes.

## Results

### Familiarity

Each participant’s ratings were used to divide the stimuli into two sets: one set consisted of brands that had the rating 1 (most unfamiliar), and the other where the rating was 5 (most familiar). Stimuli having these extreme ratings of either 1 or 5 accounted for 82.28% of the stimuli across participants (SD = 12.33%). A set of paired-sample t-tests was used to compare GNG performance for familiar and unfamiliar brands. Differences in accuracy on the GO trials (percentage of trials in which participants correctly pressed a key on a GO trial) were significantly different for the two sets of stimuli t(27) = 2.039, p = 0.051, MD = 0.714, SE = 0.350 indicating that participants were (perhaps surprisingly) *less* accurate for familiar stimuli (Fig 1a). In other words, when they saw a familiar stimulus to which they should have responded, they occasionally failed to make this response. Average reaction times for the GO trials showed no significant difference as a function of familiarity, t(27) = 0.783, p = 0.44, MD = 1.66, SE = 2.76, (Fig 1c). Performance on NOGO trials (where participants were required to inhibit response) revealed no significant difference in accuracy between conditions, t(27) = -1.33, p = 0.194, MD = -2.31, SE = 2.14, (Fig 1b). However, the reaction times for commission errors (CE, mistakenly pressing a key when a response should have been inhibited) revealed a significant difference between the stimulus sets, t(27) = 4.699,p < .001, MD = 21.70, SE = 4.619 (See Fig 1d). Specifically, participants made more rapid errors to familiar stimuli. In other words, when a stimulus repeated itself, and the participant mistakenly responded to the second occurrence, they made this error more rapidly for familiar stimuli.

Because performance on this task will be influenced by both sensitivity and criterion shifts, we next used a signal detection analysis on the accuracy data. We calculated measures of sensitivity (d’) and response bias criterion (c) [29]. These values were calculated by taking into account “hits” (responding on a GO trial) and “false alarms” (CE i.e. incorrectly responding on a NOGO trial). We found significant differences on the measure of sensitivity (d’) as a function of stimulus familiarity, t(27) = 2.523, p = 0.016, η2 = 0.84. Specifically, participants had greater perceptual sensitivity towards the *un*familiar stimuli. The criterion (c) showed no significant difference between stimulus sets, t (27) = 1.080, p = 0.290, η2 = 0.50.

We conducted further analysis to assess any effects that might have developed over time. To do this, we analyzed GO trial accuracy and CE percentage between the 1^st^ half and the 2^nd^ half of the experiment. We conducted separate repeated measure ANOVAs with factors of familiarity (two levels) by block (two levels). For GO trial accuracy there was still the main effect of familiarity [F (27,1) = 14.357, p = 0.001] however, there was no significant difference across block [F (27,1) = .702, p = 0.409], and there was no overall interaction [F (27,1) = 2.844, p = 0.103], For CE there was an effect of block [F (27,1) = 21.656, p = 0.000], meaning that as time went on participants made more CE. There was no main effect of familiarity [F (27,1) = .192, p = 0.665], or any overall interaction [F (27,1) = 3.027, p = 0.093],

### Liking

For each participant their rating was used to divide the stimuli into two sets, one where the rating was 1 (dislike), and the other where the rating was 5 (liked). These extreme stimuli comprised 74.56% of the stimuli across participants (SD = 16.88%). A set of paired-sample t-tests was used to compare liked and *dis*liked brands. GO trial accuracy was significantly higher for *dis*liked stimuli, t (27) = 2.095, p = .046, MD = .797, SE = .381, (Fig 1a). Similar to the pattern we saw with the familiarity data, this suggests that participants were more likely to fail to respond to their *liked* stimuli—perhaps suggesting an overactive inhibition for such stimuli. Reaction times on GO trials showed no significant difference across levels of liking, t (27) = -.600, p = .554, MD = -1.66, SE = 2.76, (Fig 1c). The number and speed of CEs were not significantly different across levels of liking (p<0.05) (Fig 1b and 1d).

Unlike what we found with the familiarity analysis (where d’ was higher for the *un*familiar stimuli), here the d’ measure showed no significant differences between the stimulus categories, t (27) = 1.207, p = .238, η2 = 0.78. However c showed significant differences, t (27) = 2.147, p = .041, η2 = 0.57, this suggests that there was a response bias towards the disliked (vs. liked) brands, meaning that participants were more likely to respond when viewing disliked items in comparison to liked items.

We again conducted repeated measure ANOVAs for accuracy and RTs with factors of block and liking. Apart from the main effect of liking (as above), there were no effects of block present nor any significant interactions.

#### Correlation and Brand Analysis

A Spearman’s correlation was conducted between familiarity and liking which showed a significantly strong correlation, r = 0.71, p = .000. That is the greater the rating in familiarity the greater the rating in liking. Further analysis was done on each brand by CE to see if there were any possible differences, other than familiarity and liking, between brands that had few errors and those that had the most (e.g. colour, type of brand, typography) qualitatively there seemed no apparent differences (See S1 Text **Individual Brand Analysis**.).

## Discussion

We found that familiarity and liking produced significant and distinct effects on inhibitory control for brand stimuli. To our knowledge this is the first use of brand stimuli in an inhibitory control task, and the novel findings have both theoretical and applied implications for stimulus processing and brand-related inhibitory control.

We found that participants were more likely to *erroneously* withhold responding to highly familiar stimuli. We also found an overall greater sensitivity towards unfamiliar stimuli. Could these results be explained in relation to predominate models of inhibitory control?

First we consider Logan & Cowan’s [30] horse-race model of response inhibition. Their model [30] was initially developed to explain results from the stop-signal paradigm (a conceptually similar paradigm to Go No/Go) and stipulates two processes (respond/inhibit) that compete for dominance on each trial. In this “winner takes all” model, the final behavioural outcome (respond/inhibit) will depend on whichever process passes its’ threshold first.

Previous research suggests a tendency towards approach-related (respond) behavior with positive stimuli and a tendency towards withdrawal-related (inhibit) behavior with negative stimuli (e.g. [31]). In the context of the horse-race model, this suggests a faster “respond” signal in the presence of a liked stimulus. In terms of error rates, we saw a decreased approach-related behavior to the *familiar* and *liked* stimuli, i.e. making responses towards the stimuli. We found evidence for this counter-intuitive effect in both the GO accuracy for familiar stimuli and in the lower response criterion for liked stimuli. One possible explanation for this counter-intuitive effect is that participants may have a tendency to focus their attention and behavior towards negatively valenced stimuli—a phenomenon seen in numerous behavioral tasks. That is, valance of the present stimuli create a weak level of saliency in comparison to more naturalistic and as such generally more salient stimuli.

How might familiarity impact the horse-race model processes? Recall that we observed faster reaction times for CE when the stimulus was highly familiar. In terms of the horse-race model, this seems to suggest that the respond processes was sped up. However, if this were the case, this same speeding should have also manifested itself as a difference in the GO RTs, and accuracy—a result demonstrated by several previous studies using GNG and emotional stimuli [32] but a result that we did not observe.

We found that participants were more likely to *erroneously* withhold responding to highly familiar stimuli. We also found an overall greater sensitivity towards unfamiliar stimuli. These results are particularly surprising given the simple and mind-numbing nature of the task (whereby participants should respond to 90% of the stimuli they see—once each second—for 20 minutes). We suggest that this could be due to the task demands and context effects present in the task. Specifically, in the GNG task participants are likely to be looking for a “signal to withhold responding”. They know that the (explicit) signal to withhold is the repetition of a stimulus. In other words, they must be constantly monitoring for a “repeat”. Although this may sound like a fairly straightforward task (“was this a repeat?”) it does place certain demands on the participant’s cognitive and attentional resources. As each new stimulus appears, they must decide whether this new thing is the same as the previous thing. Although this decision is achievable via reliance on traditional memory processes, perhaps within the context of the experiment, another (perhaps even easier) way to render this judgment is based on how familiar the stimulus seems. If a stimulus was *just* seen moments before, then it should be highly familiar. It might be this mechanism (which is likely non-linguistic and perhaps irrational) that leads to failures with the highly familiar stimuli. Specifically, when a highly familiar stimulus appears, the participant mistakenly attributes the immediate sense of familiarity engendered by the stimulus to a mis-remembered prior presentation of the stimulus, and thus they fail to respond a form of misattributed immediate familiarity.

Our original hypothesis was that highly familiar brand logos would have a greater “draw” and would thus make it more difficult for participants to inhibit their responding when required (i.e. more errors of commission). However, in terms of percentage of CE, we found no difference between the familiar and unfamiliar stimuli. This seems to suggest that participants are equally able to withhold responding to both classes of stimuli—in other words, they are able to successfully inhibit their responding even to highly familiar stimuli. This might suggest that, contrary to our original hypothesis, familiar brand logos have no more power/draw than unfamiliar ones. However, the story turned out to be more complex. While participants *were* equally able to withhold responding to both types of stimulus, we found that when they *did* make an CE, they made it far faster to the highly familiar stimuli. Taken together, these results suggest that whilst the “draw” of familiar/liked stimuli can be inhibited, such inhibition requires greater suppression of the highly-familiar/liked stimuli and that when this inhibition fails, the greater draw of the highly-familiar/liked stimuli is revealed via quicker errors.

Research into the déjà-vu effect [33] may provide insight into the findings of the current study. An illusion of memory (perhaps related to familiarity) can be formed by the previous unconscious processing of stimuli [34]. Within this task one of the main findings was that high familiarity created poorer performance in the GO trials, something unintuitive at first. However, considering the task requirements (i.e. to recall if the previous stimulus is repeated), the illusion of memory towards familiar brands could directly interfere with this primary task. That is, if a participant sees a highly familiar brand then their response (or lack thereof) might be influenced by their illusory memory rather than task-relevant working memory, similar to some form of memory interference [35].

Finally, as a primary aim to investigate the interactions between liking and familiarity again we show a close bounded relationship, indeed ratings for liking and familiarity were correlated and so might suggest that the effects of one categorization were driving effects of the other. And, the reduction in GO trial accuracy to both highly-familiar and highly-liked targets does suggest that this is at least partially true. However, this cannot be the whole story as the effects of liking and familiarity on other aspects of task performance were qualitatively and quantitatively different. Specifically, participants were less sensitive (in terms of d’) to familiar logos, but had equal criterion levels for making a response (in terms of c). This might suggest that their sense of familiarity could lead to their tentative nature of responding (low d’), but that it could not really change their criterion for responding (c). In contrast, their liking of a logo did not make it particularly more salient (no change in d’), but it did make it more appetitive and thus had a lower criterion for response (c). The exact nature of, and relationship between, these effects will need to be studied in future studies.

Within the context of behavioral neuroscience this study could be repeated whilst during the acquisition of neurophysiological measures (e.g. M/EEG, fMRI), indeed there exist literature [36–38] that allows us to tacitly make sure inferences of this behavioral effect to the neural circuitry involved.

There are broader applied and theoretical implications of this study. Firstly, discovering the role of inhibitory control in consumer behaviour is essential, particularly given the increasing prevalence of compulsive shopping [39]. Understanding if inhibitory mechanisms are destabilized by the presentation of brands is crucial if there is to be future development of intervention tasks, similar to those developed by researchers in addiction (e.g. [40]). For psychological research in general this study once again highlights the necessity to control for familiarity and liking, as the former may affect the accuracy of mnemonic judgments, whilst the latter may bias performance on the basis of valence. As such, a greater level of control should be placed on these stimulus characteristics, being treated both as independent and significant factors on experimental design and findings. Furthermore, there are other numerous experimental manipulations that could be tested in the future, for example testing between groups of compulsive shoppers vs. normal shoppers.

To conclude, brand familiarity and brand liking both contribute to a participant’s inhibitory performance. A déjà-vu like effect appears to influence behaviour when a participant is very familiar with a brand. That is, one could suggest that participants knew they needed to enact inhibitory control, however they over compensated for brands with which they were very familiar. When this mechanism failed, however, their desire to respond quickly was evident. Taken as a whole, the results demonstrate that preexisting affect (liking) and familiarity can dramatically alter ones ability to exert inhibitory control, and these two effects are closely tied. Future research may help unravel the relationship between these phenomena and such important real-world behaviours as brand-loyalty, compulsive shopping, and overeating.

## Supporting Information

S1 Fig The EOC percentage by brand, the horizontal axis is arbitrary. (PNG)

S2 Fig Scatter plot showing EOC percentage on the vertical and mean liking on the horizontal axis. (PNG)

S3 Fig Scatter plot showing EOC percentage on the vertical axis and mean familiarity on the horizontal axis. (PNG)

S4 Fig Scatter plot showing mean familiarity against mean liking. A linear trendline has also been appended to the scatter with the correlation coefficient and equation displayed.
(PNG)

S1 Text Individual Brand Analysis. (DOCX)
