# Correction: Brands and Inhibition: A Go/No-Go Task Reveals the Power of Brand Influence

**DOI:** 10.1371/journal.pone.0143985

**Published:** 2015-11-23

**Authors:** The PLOS ONE Staff

In Fig 1, the legend improperly differentiates between high and low familiarity/liking. Please see the corrected Fig 1 here. The publisher apologizes for the error.

**Fig 1:**
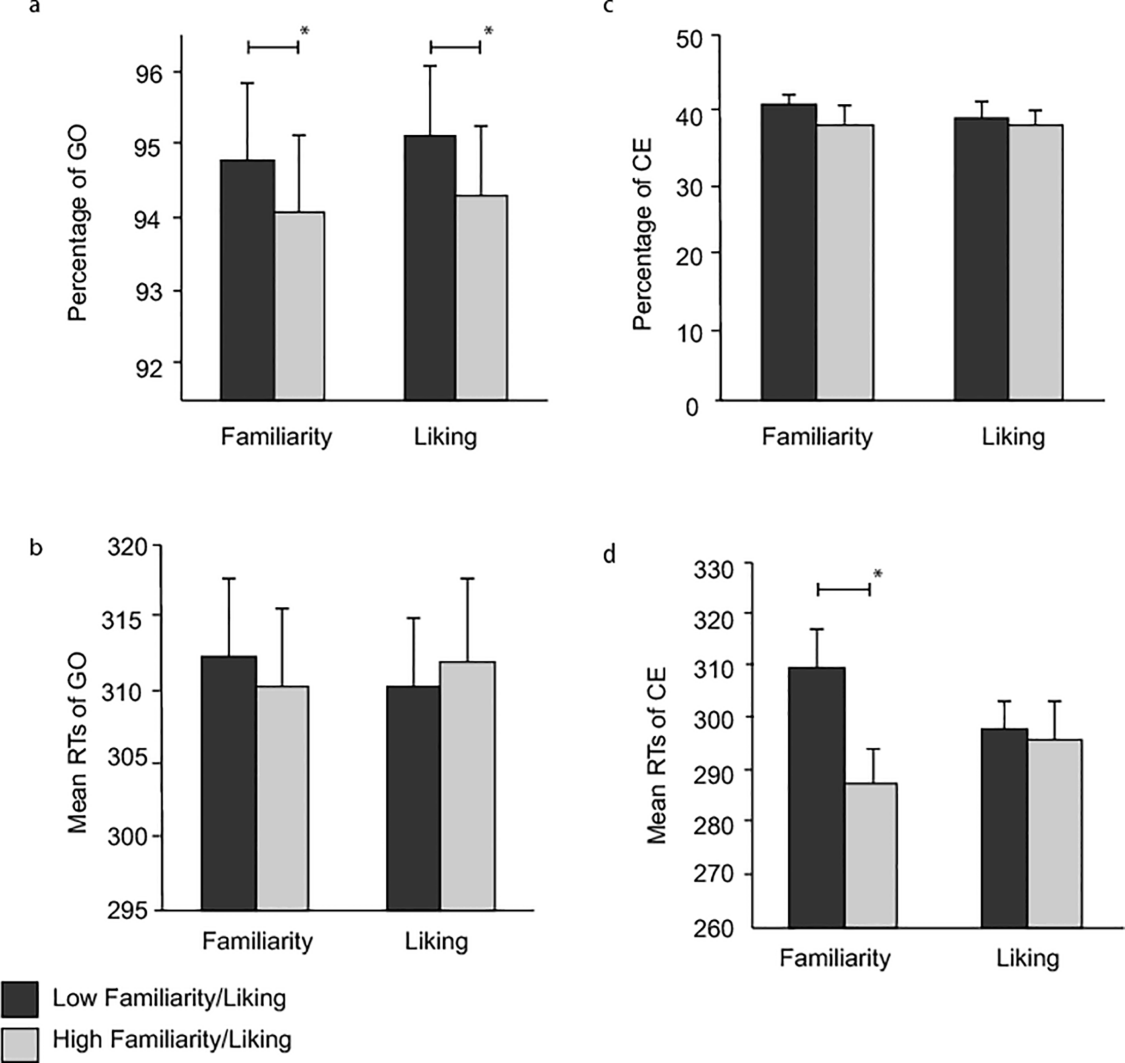
Performance of participants for the different measures, and the different subjective-ratings. (a) GO trial accuracy. I.e. pressing the space bar when required to do so. (b) Percentage of commission errors. I.e. pressing the space bar when it was not required to do so. (c) Mean RT for GO trials, (d) Mean RT for commission errors. Error bars indicate standard error. * Denotes significance.
