# Supplementary figures and images for: Brands and Inhibition: A Go/No-Go Task Reveals the Power of Brand Influence
Source: PLoS One. 2015 Nov 6;10(11):e0141787. doi: 10.1371/journal.pone.0141787 (PMC4636362; doi:10.1371/journal.pone.0141787)

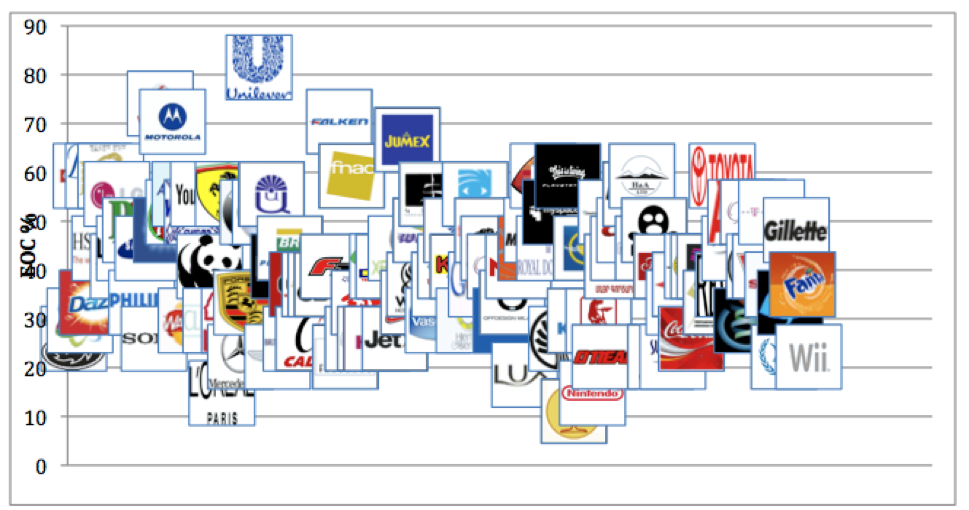

Supplement: S1 Fig — (PNG) [file pone.0141787.s001.png]

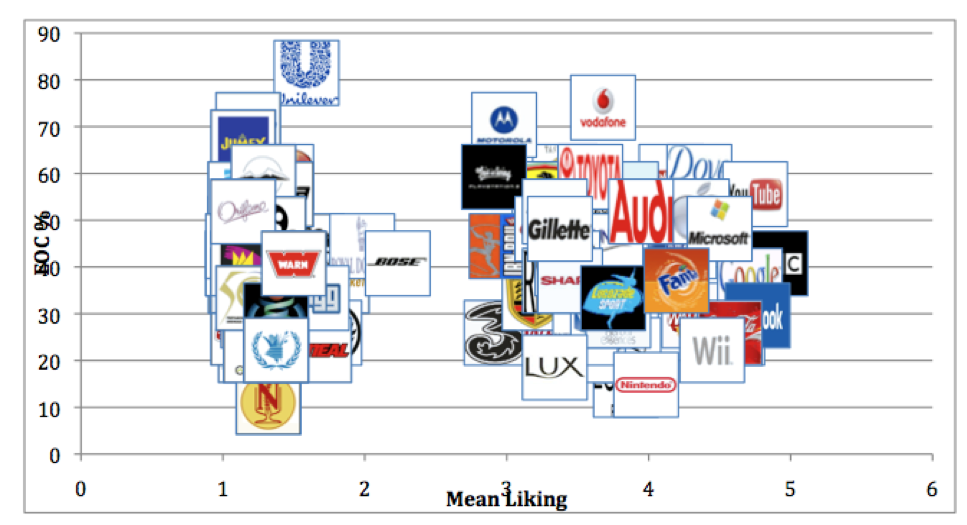

Supplement: S2 Fig — (PNG) [file pone.0141787.s002.png]

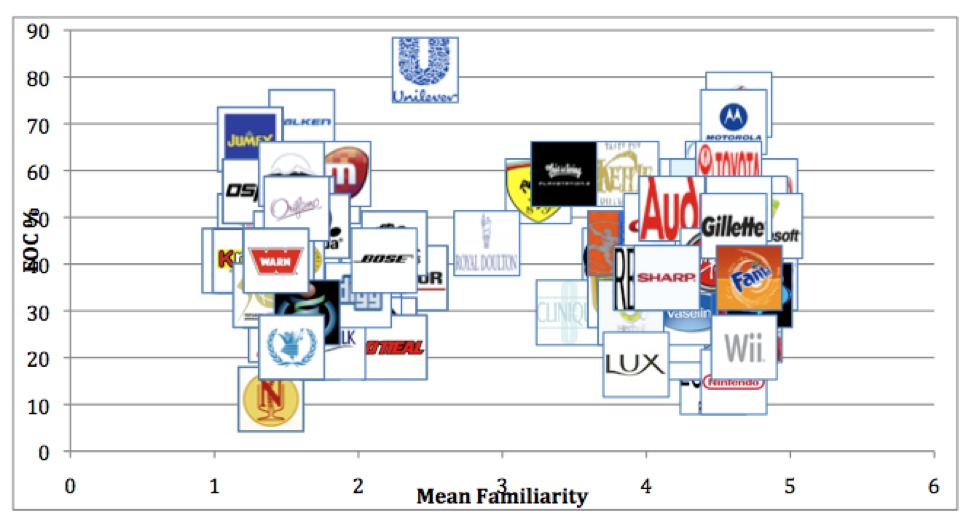

Supplement: S3 Fig — (PNG) [file pone.0141787.s003.png]

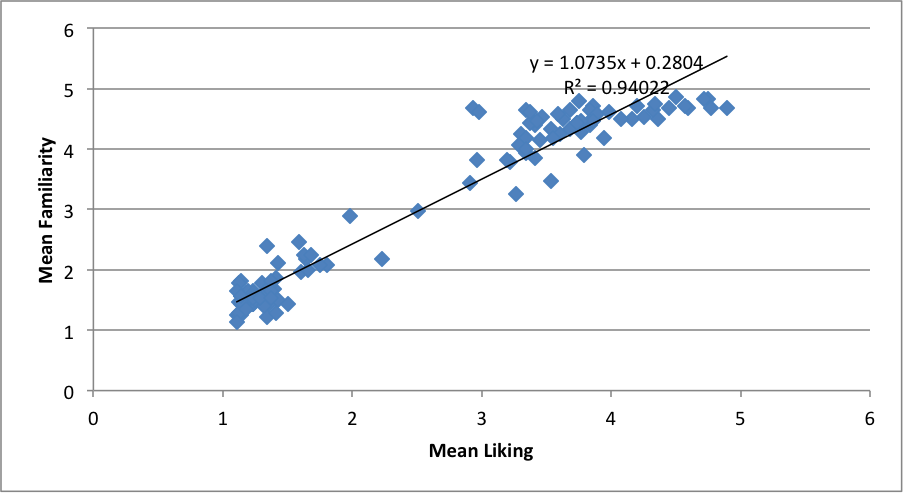

Supplement: S4 Fig — A linear trendline has also been appended to the scatter with the correlation coefficient and equation displayed. (PNG) [file pone.0141787.s004.png]
