# Supplementary material for: Brands and Inhibition: A Go/No-Go Task Reveals the Power of Brand Influence
Source: PLoS One. 2015 Nov 6;10(11):e0141787. doi: 10.1371/journal.pone.0141787 (PMC4636362; doi:10.1371/journal.pone.0141787)
Supplement: S1 Text — (DOCX) [file pone.0141787.s005.docx]

**S1 Text. Individual Brand Analysis.**

Correlation and Brand Analysis

A Spearman’s correlation was conducted between familiarity and liking which showed a significantly strong correlation, r = 0.71, p = .000. That is the greater the rating in familiarity the greater the rating in liking. Further analysis was done on each brand by EOC to see if there were any possible differences, other than familiarity and liking, between brands that had few errors and those that had the most (e.g. colour, type of brand, typography) qualitatively there seemed no apparent differences.

Brand Analysis – These graphs can be removed from the final submission, as they do not really add any information at all.

For each brand image we plotted a number of scatter graphs to examine the mean EOC percentage (Fig. S1), familiarity (Fig. S2), and liking (Fig. S3), and finally familiarity vs. liking (Fig. S4). Added to this we conducted analysis on the two rating dimensions (Table S1).

**Fig. S1.** The EOC percentage by brand, the horizontal axis is arbitrary.

**Fig. S2.** Scatter plot showing EOC percentage on the vertical and mean liking on the horizontal axis.

**Fig. S3.** Scatter plot showing EOC percentage on the vertical axis and mean familiarity on the horizontal axis.

**Fig. S4.** Scatter plot showing mean familiarity against mean liking. A linear trendline has also been appended to the scatter with the correlation coefficient and equation displayed.
